# Promising sensors for pharmaceutical pollutant adsorption using Clar’s goblet-based 2D membranes

**DOI:** 10.1038/s41598-023-50802-0

**Published:** 2024-01-09

**Authors:** Mahmoud A. S. Sakr, Mohamed A. Saad, Omar H. Abd-Elkader, Hazem Abdelsalam, Qinfang Zhang

**Affiliations:** 1 Chemistry Department, Center of Basic Science (CBS), Misr University of Science and Technology (MUST), 6th October City, Egypt; 2 Physics Department, Center of Basic Science (CBS), Misr University of Science and Technology (MUST), 6th October City, Egypt; 3 Department of Physics and Astronomy, College of Science, King Saud University, P.O. Box 2455, 11451 Riyadh, Saudi Arabia; 4 Theoretical Physics Department, National Research Centre, El-Buhouth Str., Dokki, Giza, 12622 Egypt; 5 School of Materials Science and Engineering, Yancheng Institute of Technology, Yancheng, 224051 People’s Republic of China

**Keywords:** Chemistry, Materials science, Physics

## Abstract

This study focuses on the design of new 2D membranes from connected Clar’s Goblet as a potential sensor for pharmaceutical pollutants, specifically the painkiller drugs aspirin, paracetamol, ibuprofen, and diclofenac. The electronic, optical, and interaction properties are investigated using density functional theory calculations. The Clar’s Goblet membranes (CGMs) that were chosen are semiconductors with an energy gap of around 1.5 eV, according to energy gap calculations and density of states. Molecular electrostatic potential (ESP) analysis shows that CGMs have electrophilic and nucleophilic sites, suggesting their suitability for interacting with pharmaceutical pollutants. The adsorption energies confirm the chemical adsorption of pharmaceutical pollutants with diclofenac showing the strongest adsorption. The UV–Vis absorption spectra of CGMs-drug complexes are analyzed, revealing a redshift compared to the absorption spectrum of CGMs alone, confirming the adsorption of these drugs. Further analysis using hole/electron examinations indicates that the type of excitation is local excitation rather than charge transfer excitation. This study quantitatively characterized hole and electron distribution in excited states using various indices. The analysis revealed local excitation transitions and significant charge transfer between the CGMs molecule and pharmaceutical pollutants. Additionally, non-covalent interaction analysis indicates the presence of van der Waals interactions, highlighting the adsorption behavior of the drugs. These results demonstrate the potential of CGMs as a highly sensitive sensor for pharmaceutical pollutants.

## Introduction

Two-dimensional (2D) materials, such as graphene, transition metal dichalcogenides, and black phosphorus, have gained significant attention due to their unique properties and potential applications^1^. Graphene, for example, is a thin sheet of carbon atoms arranged in a honeycomb lattice and exhibits excellent mechanical, electrical, and thermal properties^2^. These properties make it a promising material for various applications, such as electronics, energy storage, and sensors^3^. Similarly, transition metal dichalcogenides, such as molybdenum disulfide and tungsten diselenide, have emerged as promising candidates for electronic and optoelectronic applications due to their direct bandgap and strong light-matter interactions^4^. Black phosphorus, on the other hand, exhibits anisotropic properties and has shown promise in applications such as transistors, photodetectors, and energy storage devices^5^. The unique properties of 2D materials make them promising candidates for a wide range of applications, and research in this field is rapidly advancing.

Two-dimensional (2D) quantum dots, which are essentially 2D nanocrystals, have been extensively studied due to their unique size-dependent properties and potential applications in optoelectronics and photonics^6,7^. These quantum dots exhibit quantum confinement in two dimensions, resulting in discrete energy levels and tunable optical properties^8,9^. Unlike their three-dimensional counterparts, 2D quantum dots have a larger surface-to-volume ratio, which enhances their surface chemistry and allows for greater control over their optical and electronic properties^10,11^. Additionally, 2D quantum dots can be easily integrated with other 2D materials, such as graphene and transition metal dichalcogenides, for use in hybrid nanodevices^12–14^. Research in this field is rapidly advancing, and 2D quantum dots hold great promise for applications in areas such as solar cells, light-emitting diodes, and quantum computing.

Two-dimensional (2D) porous materials, such as graphene oxide, have shown great potential for use in wastewater treatment due to their high surface area, tunable pore size, and excellent adsorption capabilities^15^. Graphene oxide membranes have been used for the efficient removal of heavy metal ions, organic dyes, and pharmaceuticals from wastewater^16^. Additionally, 2D covalent and metal–organic frameworks have emerged as promising materials for the removal of contaminants from water due to their high porosity and surface area^17,18^. They can be easily synthesized with desired chemical properties and pore sizes, allowing for selective adsorption of specific contaminants from wastewater^19^. These materials have also been used in the development of membrane technology for wastewater treatment, with the potential for scalable production and application^20^. Moreover, 2D quantum dots or nanoribbons can be connected to each other to form 2D membranes with abundant active sites and controllable pore size^21–24^. For instance, Abdelsalam et al. show theoretically that nanoporous graphene membranes constructed from triangulene are potential candidates for the separation of petroleum hydrocarbons^24^. These triangulene (zigzag-triangular graphene quantum dots) and graphene nanostars have been already synthesized^25–28^. Therefore, 2D porous materials hold great promise for the efficient and selective removal of contaminants from wastewater, and research in this field is rapidly advancing. Graphene and graphene oxide (GO)^29^ played crucial roles in diverse research fields, particularly in the development of porous graphene materials (PGMs). The investigation covered the chemistry of graphene functionalization, the creation of varied pore structures, and the potential applications of PGMs in energy storage, electrocatalysis, and molecular separation, highlighting the essential requirement for precise control over pore morphology and size to achieve optimal performance.

Clar’s Goblet (CG) is a nanographene consisting of fused aromatic rings, with two unpaired electrons and potential applications in electronics, energy storage, and catalysis^30–38^. Recent synthetic advancements have enabled the characterization of CG, making it a promising candidate for the design of new advanced materials due to its hexagonal shape and two different reactive sites. Furthermore, CG has been shown to improve charge transport properties in organic solar cells and has the potential for the development of new organic magnets due to the confinement of the two unpaired electrons within the molecule^30–37^.

Pharmaceutical pollutants, specifically painkillers like ibuprofen and acetaminophen, are a growing environmental concern due to their presence in wastewater treatment plants and natural water systems and their potential to harm aquatic life and human health. These pollutants are not entirely removed during wastewater treatment and their continuous release can lead to antibiotic-resistant bacteria, presenting a challenge to public health. Hence, it is essential to develop effective methods for their removal from wastewater and monitor their occurrence and transportation in natural water systems^39,40^. This study introduces a novel solution to the increasing environmental concern of pharmaceutical pollutants, particularly painkillers such as aspirin, paracetamol, ibuprofen, and diclofenac, which are commonly detected in natural water systems. The researchers designed porous 2D membranes based on Clar’s Goblet, named Clar’s goblet membranes (CGMs), to function as a highly sensitive sensor for these pollutants. The study investigates the electronic, adsorption, optical, and interaction properties of CGMs with these drugs, estimating the adsorption energy and electronic energy gap. The results reveal a redshift in the absorption spectra of CGM1-drug complexes, confirming the absorption of these painkillers on the surface of CGMs. The study's findings suggest that CGMs have the potential to be an efficient and reliable sensor for pharmaceutical pollutants, with possible applications in wastewater treatment and environmental monitoring.

## Computational methodology

The electronic and optical properties of CGMs before and after the adsorption of the pharmaceutical pollutants are investigated using density functional theory (DFT)^41–46^ simulations as implemented in Gaussian 16^22,24,47–50^. The hybrid B3LYP functional^51,52^ is used in this work since it has been analyzed and shown to give an adequate representation of the electronic and optical properties of C-based structures^53–56^. The chosen basis set is the 6-31G^57^, which produces results with acceptable accuracy when considering the required computational power^58–60^. The time-dependent DFT calculations for the first twenty excited states are used to analyze the optical properties. The Non-covalent interaction (NCI) analysis^61^, charge transfer, and hole/electron investigations are calculated using Multiwfn software^62^ and visual molecular dynamics (VMD)^63^. The adsorption energy (E_ads_) was determined through the computation of the energy difference between the CGM/pharmaceutical pollutant complex (E_CGM/pharmaceutical pollutant_) and the sum of individual energies of the CGM compound (E_CGM_) and the pharmaceutical pollutant (P_harmaceutical pollutant_). Mathematically, this is expressed as: *E*_ads_ = *E*_CGM/pharmaceutical pollutant_ − (*E*_CGM_ + *E*_pharmaceutical_ _pollutant_)^18,24,49^.

## Results and discussions.

### Optimized molecular structures

**Figure 1:**
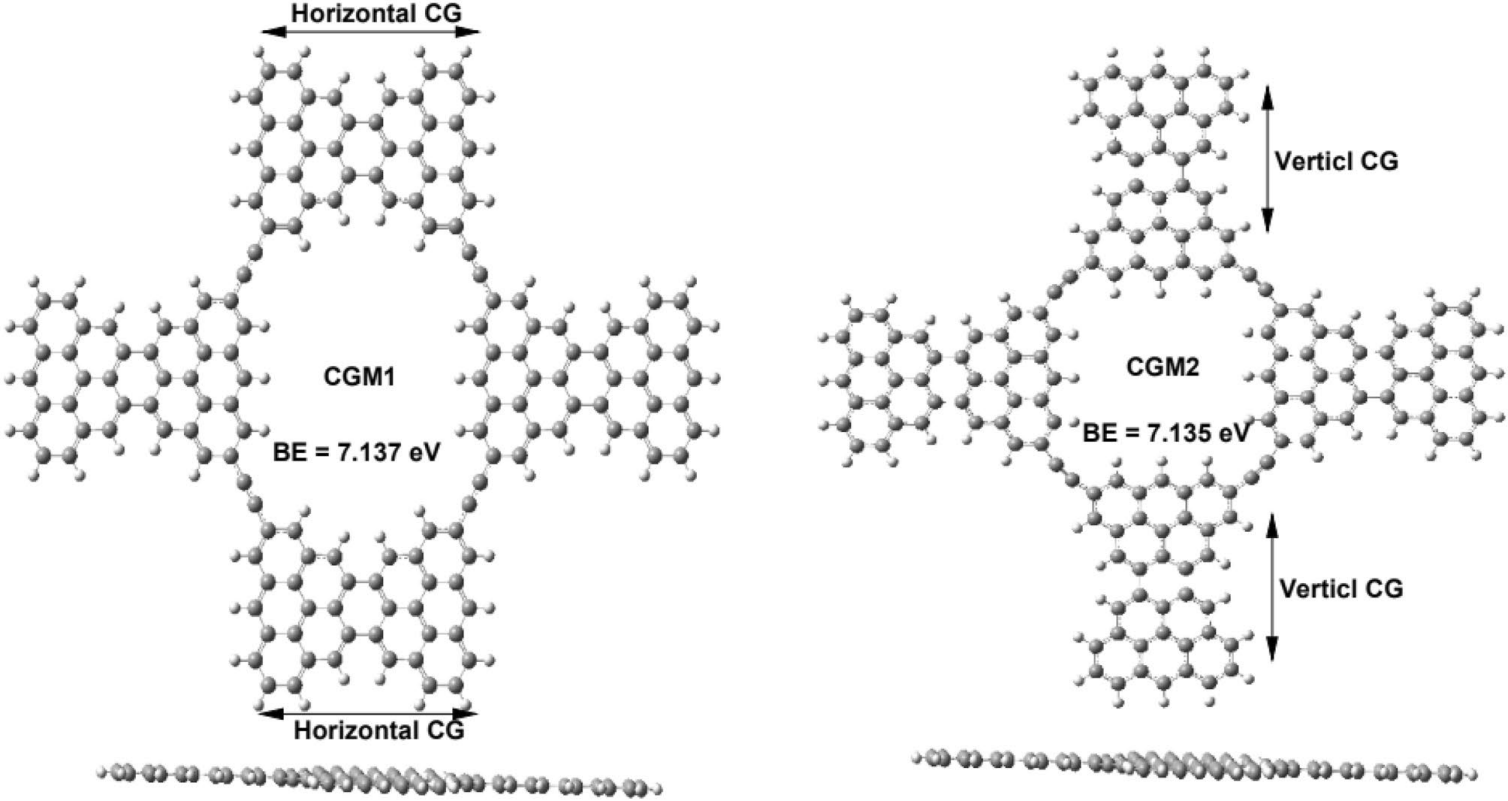
Optimized molecular structures of CGM1 and CGM2 in both front and side views. The optimization was performed using the B3LYP/6-31G level of theory in the gaseous state.

Figure 1 illustrates the optimized molecular structures of Clar’s goblet membranes (CGMs), which are composed of CG units connected through acetylene bridges to form a porous structure. The study investigates two forms of CGMs, namely CGM1 and CGM2. The distinguishing factor between these forms is the type of interaction between the CG units. In CGM1, the above and below CG units are horizontal, while in CGM2, they are vertical. However, both CGM1 and CGM2 have vertical left and right CG units. These structural variations affect the binding energy and the internal edge carbons of the material, which can influence its sensor properties towards pollutant materials. Both CGM1 and CGM2 are made up of carbon atoms passivated with hydrogen atoms, as depicted in Fig. 1. The optimized molecular structures of CGM1 and CGM2 are displayed in Fig. 1, which allowed for the determination of the optimal bond length, bond angle, and dihedral angle. The C–C bond lengths range from 1.0828 Å to 1.4794 Å, while the C–C–C bond angles range from 116.65° to 122.78°, indicating that the type of hybridization is sp^2^. The C–C–C–C dihedral angle is around 180.00 degrees, and the side views suggest that the surface of the CGMs is flat. To calculate the binding energy (BE) per unit atom, the formula BE = (N_C_E_C_ + N_H_E_H_—E_t_)/N_t_^18,22,24,48–50^ was utilized, where N_C_, N_H_, and N_t_ represent the number of carbon atoms, number of hydrogen atoms, and the total number of atoms in the system, respectively. The total energies of the carbon atoms (E_C_), hydrogen atoms (E_H_), and the resultant compound (E_t_) were used in the calculation, and the BE values obtained are displayed in Fig. 1. Positive values of BE indicate that the considered CGMs systems are stable. It is worth noting that the BE value of CGM1 is higher than that of CGM2, indicating that the molecular structure of CGM1 is slightly higher stable compared to CGM2.

### Electronic properties

**Figure 2:**
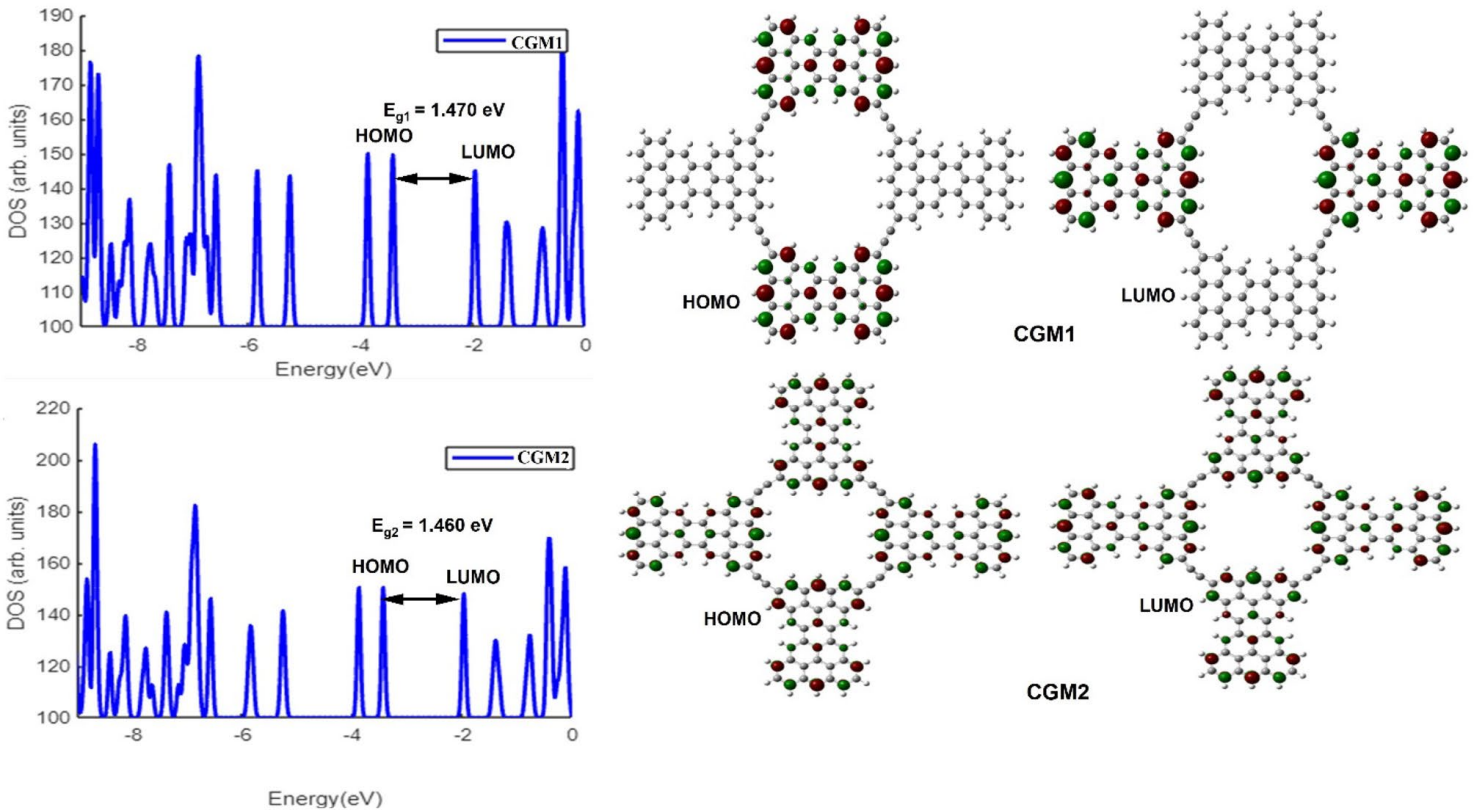
Electronic density of states of different CGM and the corresponding HOMO and molecular orbitals.

The energy gap (E_g_) between the highest occupied molecular orbital (HOMO) and the lowest unoccupied molecular orbital (LUMO) is a crucial factor in understanding the electronic properties of a system^64^. The HOMO describes the electron-donating ability of a molecule, while the LUMO describes its electron-withdrawing ability. Figure 2 shows a graphical representation of the HOMO and LUMO orbitals for CGM 1 and CGM2. In CGM1, the HOMO is localized on the horizontal CG groups above and below the molecule, while the LUMO is localized on the CG units to the left and right of the molecule. This indicates that the horizontal CG units donate electrons to the vertical CG units in CGM1. However, in CGM2, all CG units are vertical, so the HOMO and LUMO are localized over the entire molecule except for the acetylene bridge. The energy gap values for both CGM1 and CGM2 were calculated in the gaseous state and are shown in Fig. 2. This suggests that when two CG units are vertical and two are horizontal, the CGM is slightly higher stable than when all CG units are vertical. The electronic density of states (DOS) for CGM 1 and CGM2 compounds is also shown in Fig. 2, with energy levels obtained using a Gaussian function $\frac{1}{\alpha \sqrt{2\pi }}{\text{exp}}[-\frac{{(\in -{\in }_{i})}^{2}}{2{\alpha }^{2}}]$ with broadening $\alpha = 0.1\,{\text{eV}}.$ It is evident from the figure that the energy gap slightly depends on the shape of the CGMs molecule.

### ESP investigations

**Figure 3:**
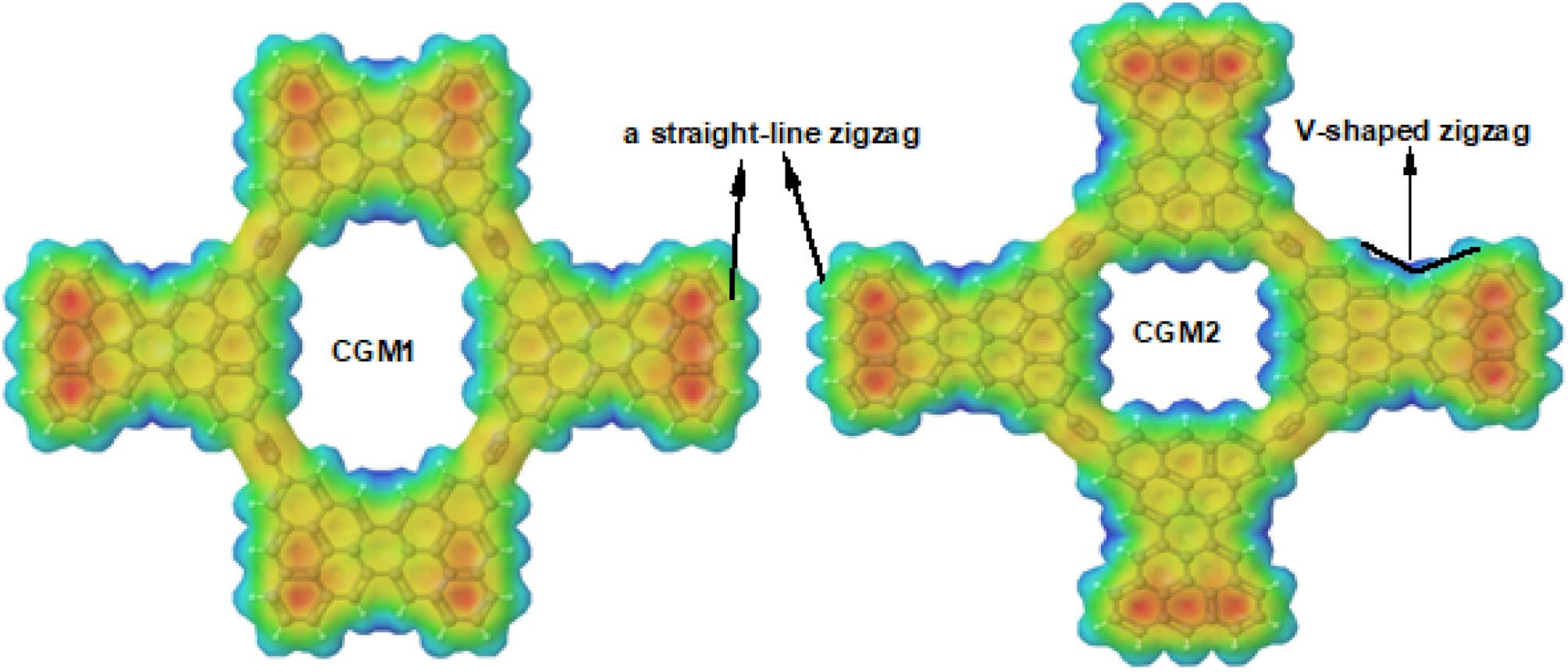
ESP of CGM1 and CGM2 in the gaseous state.

Molecular electrostatic potential (ESP) analysis is a useful tool for predicting reaction sites in chemical systems. In this study, ESP analysis was performed on the designed CGM1 and CGM2 molecules to identify their electrophilic and nucleophilic sites. The ESP surfaces are presented in Fig. 3, where the red color indicates an electron-rich, partially negative charge, the blue color represents an electron-deficient, partially positive charge, and yellow represents an electron-rich, slightly positive region. The presence of a negative region inside the terminal hexagonal rings (red color) suggests that these sites are prone to electrophilic attacks. Conversely, as all the hydrogen atoms are in the positive region (blue color), nucleophiles are likely to target these sites. The molecular structures investigated in this study showed that the hexagonal rings situated on the outer boundaries exhibit higher negative electronic densities compared to those on the inside. Comparing the edges of both compounds under study, we find that one edge takes the form of a straight-line zigzag while the other end takes the form of a V-shaped zigzag. We can see that the straight-line zigzag form has a higher density of negative charge than the other form, as shown in Fig. 3. Overall, the ESP analysis suggests that CGM1 and CGM2 are suitable for interactions with pharmaceutical pollutants such as aspirin, diclofenac, ibuprofen, and paracetamol that are commonly detected in water bodies such as rivers, lakes, streams, groundwater, soil, and sediment.

### CGM1 sensor

**Figure 4:**
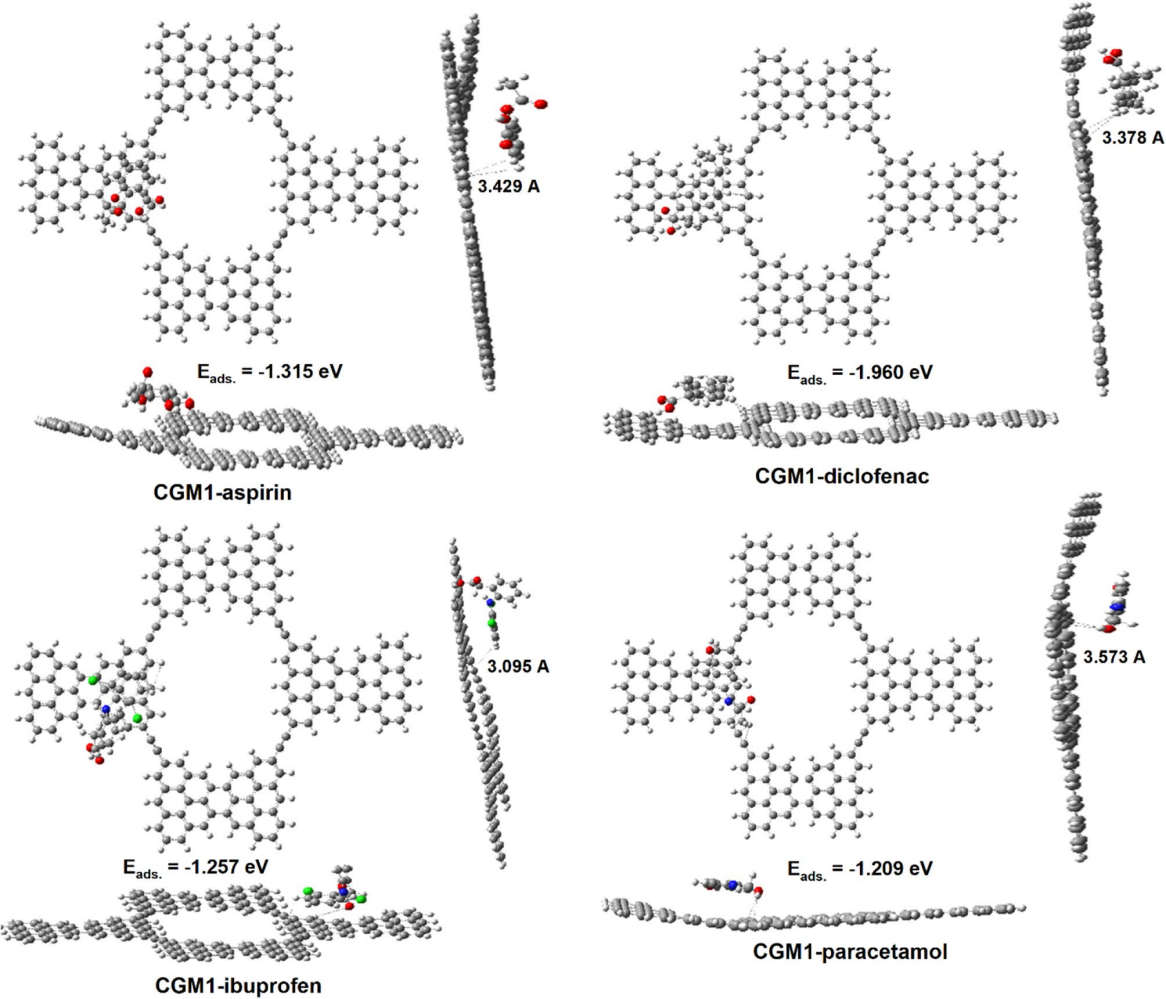
Optimized structures of CGM1-pharmaceutical pollutant drugs.

CGM1 was selected to act as a sensor with the following pharmaceutical pollutants: aspirin, diclofenac, ibuprofen, and paracetamol due to its high binding energy which is important for enhancing membrane reusability. The optimal structures for CGM1-aspirin, CGM1-diclofenac, CGM1-ibuprofen, and CGM1-paracetamol are displayed in Fig. 4. The bonding lengths and the adsorption energy (E_ads_) between pharmaceutical pollutants and CGM1 are also shown. The bond lengths between painkiller substances and the surface of CGM1 are in the range between 3.059 and 3.573 Å. There is a small deformation for the MCG 1 under research after adsorption, as illustrated in Fig. 4 (see horizontal and vertical view sides). This information indicates that CGM1 can function as a pharmaceutical pollutant sensor. The negative sign of E_ads_ validated the stability of CGM1-aspirin, CGM1-diclofenac, CGM1-ibuprofen, and CGM1-paracetamol. Adsorption energy is frequently used in studies to distinguish between chemical and physical adsorption^65^. If the adsorption energy value is between − 0.311 and − 0.104 eV^66^, the reaction is classified as physical adsorption^61^. Otherwise, if the value is between − 9.949 and − 0.518 eV^66^, chemical adsorption is taking place. The E_ads_ values for CGM1-pharmaceutical substances at the surface fall between − 1.029 and − 1.960 eV, as shown in Fig. 4. Thus, all the investigated pharmaceutical pollutants are chemically adsorbed to the surface of CGM1. According to Fig. 4, The order of decreasing negative adsorption energy values is as follows: CGM1-diclofenac has the highest value, followed by CGM1-aspirin, CGM1-ibuprofen, and finally CGM1-paracetamol. Because of this, diclofenac is adsorbed more strongly on the surface of CGM1 than other pharmaceutical pollutants.

In assessing potential reviewers for the examination of pharmaceutical pollutants such as aspirin, diclofenac, ibuprofen, and paracetamol commonly detected in water bodies, it is crucial to conduct adsorption calculations within the water phase as the solvent. Among these compounds, aspirin was selected for the determination of its adsorption energy (E_ads_) on CGM1 in water. The calculated E_ads_ for aspirin in water was found to be − 1.27 eV, with the negative value indicating the stability of the adsorption process. However, it is noteworthy that the E_ads_ value demonstrated a decrease compared to the E_ads_ of aspirin in a gas solvent (− 1.315 eV). This reduction in E_ads_ could be attributed to solute–solvent interactions, emphasizing the influence of the solvent phase on the adsorption behavior. In water, solute molecules (aspirin) interact with water molecules, and these interactions can influence the adsorption behavior. The negative E_ads_ value implies that the adsorption process is favorable and stable. In summary, based on the provided information, it can be inferred that pharmaceutical pollutants, represented by aspirin, exhibit favorable adsorption in water, and the decrease in adsorption energy compared to the gas phase highlights the role of solute–solvent interactions in influencing adsorption behavior.

### HOMO/LUMO

**Figure 5:**
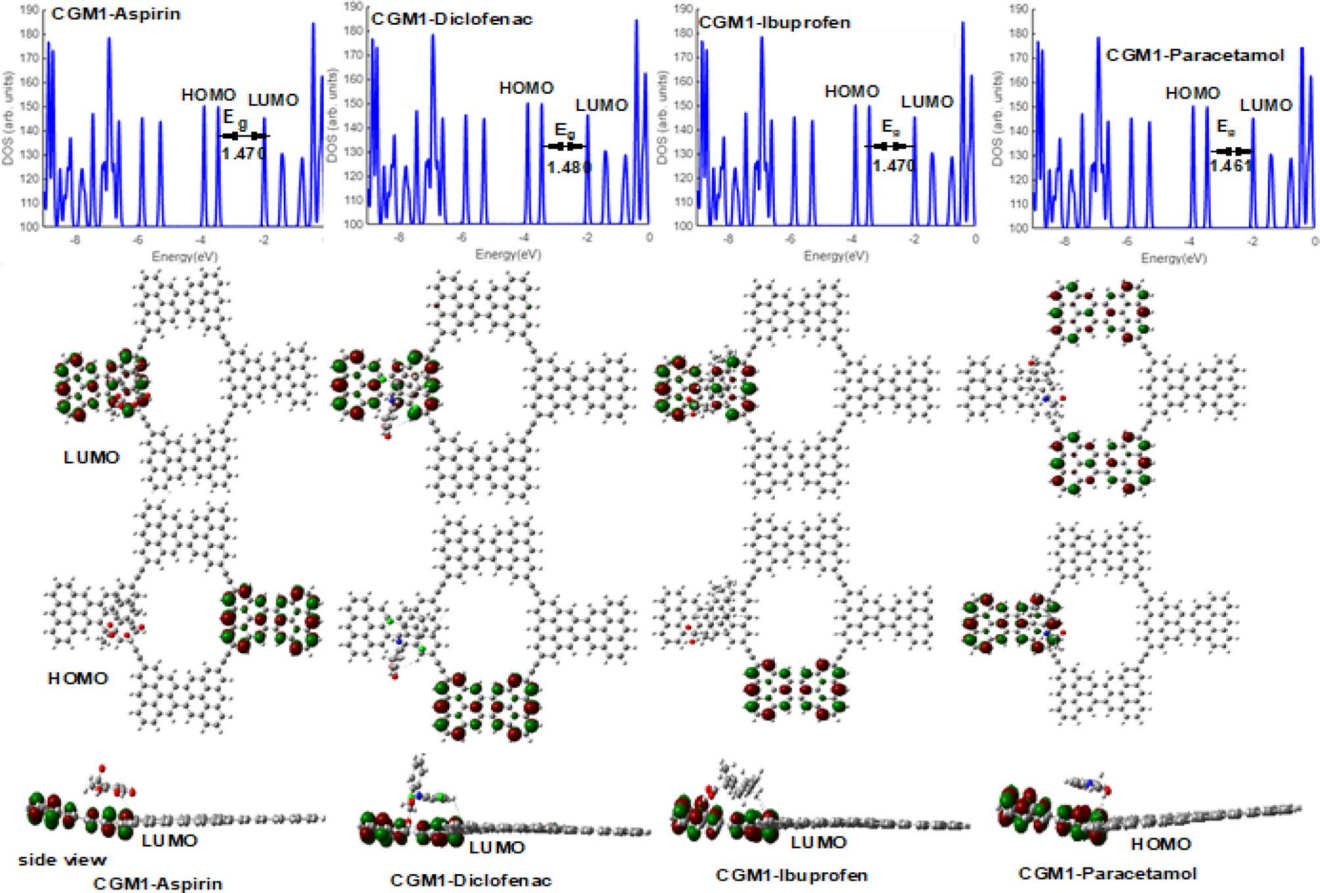
HOMO/LUMO MOs and DOS for CGM1/pharmaceutical pollutant drugs complexes.

In Fig. 5, the HOMO and LUMO orbitals of CGM1 and its complexes are graphically represented. The adsorption of different painkiller pharmaceutical drugs onto CGM1 causes a shift in the positions of the HOMO and LUMO, which depend on the adsorbed drug. For CGM1-diclofenac and CGM1-ibuprofen, the HOMO is localized on the bottom-horizontal CG group, while the LUMO is localized on the left CG units. Conversely, the HOMO of CGM1-aspirin is in the right CG unit, and its LUMO is in the left one. Finally, the HOMO of CGM1-paracetamol is located on the left CG unit, while the LUMO MOs are located at the upper and lower CG units. The adsorbed pharmaceutical painkillers do not participate in HOMO and LUMO MOs, as indicated in the side views. The energy gap values for CGM1 and its complex forms were calculated in the gaseous state and are shown in Fig. 5. CGM1–diclofenac was found to have a higher E_g_ than that of CGM1 and its complexes, indicating that it is more electronically stable than CGM1 and the other complex forms. Conversely, CGM1-paracetamol has a lower E_g_ value compared to CGM1 and the other complexes. The E_g_ values of CGM1-aspirin and CGM1-diclofenac are unchanged compared to the E_g_ value of CGM1. The adsorption of different pharmaceutical painkiller drugs onto CGM1 causes a shift in the positions of the HOMO and LUMO, depending on the adsorbed drug. This information is crucial for understanding the electronic interactions during the adsorption process. For instance: (1) Localization of HOMO and LUMO: The specific localization of HOMO and LUMO on different parts of the CGM1 molecule and adsorbed pharmaceutical drugs suggests changes in the electronic structure due to adsorption. For example, CGM1-diclofenac and CGM1-ibuprofen show HOMO localized on the bottom-horizontal CG group, while LUMO is localized on the left CG units. (2) Energy Gap (E_g_) Changes: The energy gap values (E_g_) for CGM1 and its complexes are calculated, indicating electronic stability. The variation in E_g_ values among complexes, such as higher E_g_ for CGM1-diclofenac and lower E_g_ for CGM1-paracetamol, suggests different electronic behaviors upon adsorption.

### NCI analysis

**Figure 6:**
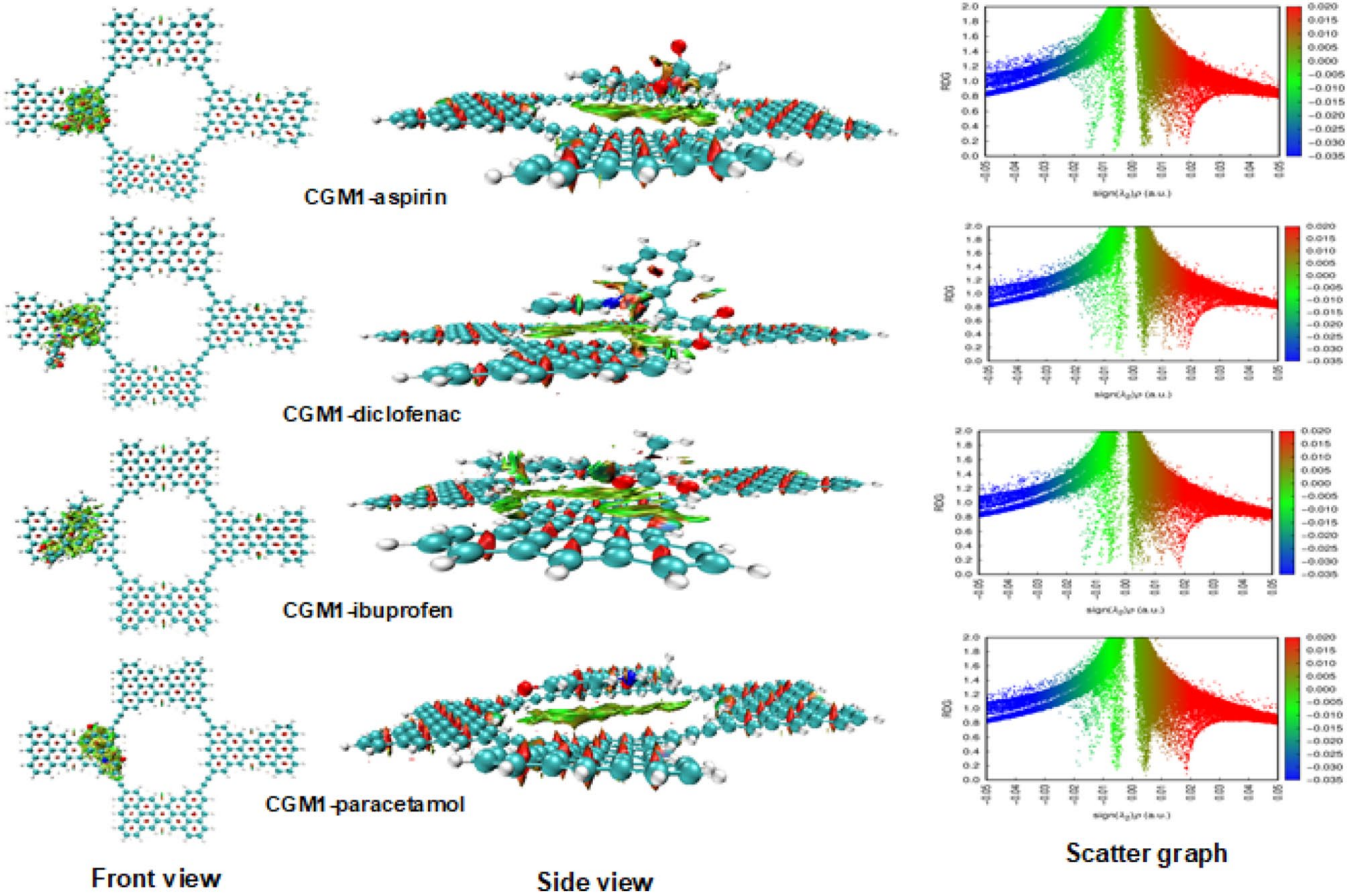
NCI surfaces (front and side views) and scatter graph (2D graph) of CGM1-aspirin, CGM1-diclofenac, CGM1-ibuprofen and CGM1-paracetamol.

Non-covalent interaction (NCI) analysis is a useful tool to study the various types of interactions such as hydrogen bonding, van der Waals forces, strong attractive forces, and repulsive steric interactions^61^. NCI analysis aims to gain insights into the nature and types of interactions. NCI analysis is presented using a two-dimensional reduced density gradient (RDG) and a three-dimensional iso-surface. The analysis provides plots of non-covalent interactions, as well as plots of RDGS against (sign λ2)ρ^67^. For strong interactions between pollutant pharmaceutical drugs and the CGM1 substrate molecule, the value of (sign λ2) ρ should be less than zero, while for repulsion of pollutant pharmaceutical drugs and CGM1, the value should be greater than zero. In the 3D NCI analysis, different color patches are used to represent different types of interactions. Yellow and green patches show van der Waals interactions, blue patches represent strong attractive interactions, and red patches signify steric repulsion. Attractive interaction points appear at a higher density value (ρ > 0.01 au), while repulsive interaction points occur at a lower density value (ρ < 0.01). The NCI analyses 3D images and their 2D plots are depicted in Fig. 6. In the case of pharmaceutical drugs adsorbed on CGM1 complexes, green patches are present between the drugs and substrate CGM1 molecules, as shown in the side and front views of Fig. 6. These complexes exhibit significant van der Waals interactions. All optimized complexes exhibit van der Waals interaction spikes at ρ < 0.01 au in the 2D graph, while repulsion interactions spike at ρ > 0.01 au. High-density green spikes appear in the region of (sign λ2)ρ values of 0.00 to − 0.01 au with green patches for drug-adsorbed CGM1 complexes, indicating that van der Waals interactions play a central role in drug adsorption.

### Optical investigations

**Figure 7:**
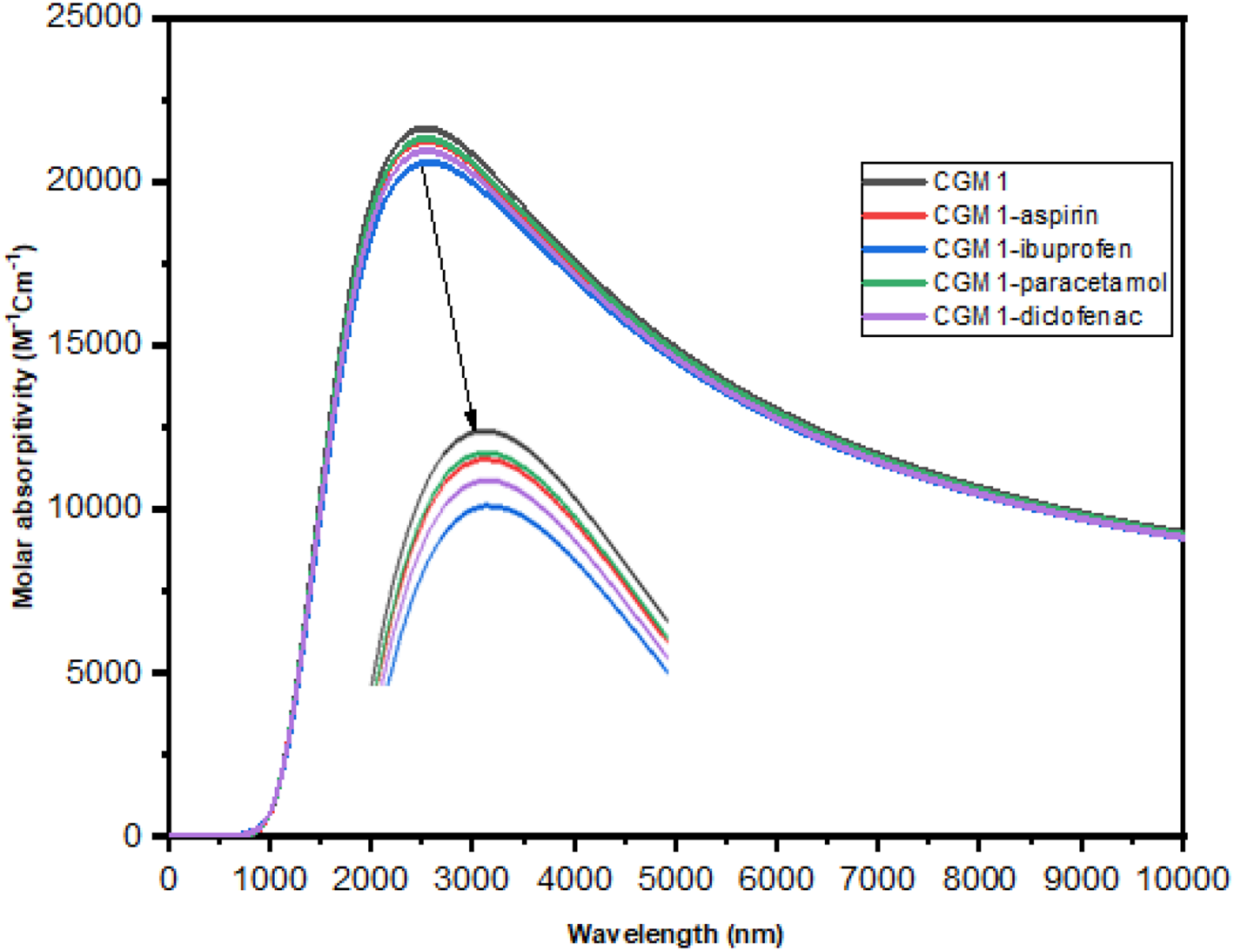
Calculated UV–Vis spectrum for CGM1 and its derivatives in the gas state.

The computed UV–Vis absorption spectra, along with relevant parameters, are presented in Table 1 and Fig. 7. The observed maximum absorbance for the studied complexes occurs due to electronic transitions from the highest occupied molecular orbital (HOMO) to the lowest unoccupied molecular orbital (LUMO). The maximum absorbance wavelengths (λ_max_) for CGM1, CGM1-aspirin, CGM1-diclofenac, and CGM1-paracetamol are 2387.00, 2409.23, 2470.16, 2472.09, and 2407.04 nm respectively. As shown in Fig. 7 and Table 1, the absorption spectra of the complexes shifted towards the red compared to CGM1, indicating the absorption of pharmaceutical painkiller pollutants on MCG 1^24,68^. Moreover, the studied pharmaceutical painkiller drugs influence the position, excitation energy (E_ex_), and oscillator strength of the absorption spectra of CGM1, as demonstrated in Fig. 7.

**Table 1:** Calculated electronic absorption of CGM1 and its based materials.

Complex	Excited state	λ_max_	E_ex_(eV)	Electronic transition	Oscillator strength
CGM1	15	2387.00	0.5021	H → L	0.5276
CGM1-aspirin	15	2409.23	0.488	H → L	0.4978
CGM1-ibuprofen	14	2470.16	0.484	H → L	0.3363
CGM1-diclofenac	14	2472.09	0.4727	H → L	0.4546
CGM1-paracetamol	15	2407.04	0.488	H → L	0.5028

### Characterization of excited states

In computational chemistry, the characterization of excited states is essential for understanding the electronic structure of molecules and their properties. Several indices have been developed to describe the nature of excited states, including Sr indices, centroid coordinate of hole and electron, D indices, H_λ_/HCT/H indices, t index, and hole and electron delocalization index. These indices provide valuable insights into the degree of charge transfer, delocalization, and spatial distribution of charge density in excited states. These indices have been widely used in computational chemistry to understand the electronic structure of molecules and their properties. Several studies have reported the use of these indices in understanding the excited state properties of different systems, such as organic molecules and transition metal complexes^69–73^. The ∆r index^74^ is used to measure the charge-transfer length during electron excitation. Local excitation (LE) refers to a situation where a hole and an electron are found nearby, while charge-transfer excitation (CT) involves significant spatial separation resulting in noticeable charge density displacement. The HDI and EDI indices are particularly useful in quantifying the breadth of their spatial distribution^75^. All hole/electron parameters are calculated using Multiwfn software^62^; the obtained results are collected in Table 2.

**Table 2:** Hole/electrons parameters.

Electronic transitions	D (Å)	Sr (au)	H (Å)	T (Å)	HDI	EDI	Δr (A)
S0 → S1	1.229	0.02678	12.687	− 5.653	3.69	3.72	0.026522
S0 → S5	0.007	0.03095	12.576	− 7.898	3.67	3.72	0.006467
S0 → S10	6.489	0.95343	11.706	− 4.281	3.82	3.88	0.018789
S0 → S15	0.000	0.99968	13.116	− 12.777	3.57	3.66	0.017367
S0 → S20	0.000	0.96566	12.573	− 10.635	2.29	2.36	0.017470

Table 2 provides data on various indices such as D, Sr, H, t, Δr, hole delocalization index (HDI), and electron delocalization index (EDI) for all twenty excited states calculated in the CGM molecule system. To select representative states, we have chosen five excitation states, namely S1, S5, S10, S15, and S20. Based on the Δr indices, we can infer that the transitions from the ground state (S0) to the excited states S1, S5, S10, S15, and S20 are predominantly local excitation (LE) excitations because their Δr indices are significantly low, with a suggested criterion of 2.0 Å to distinguish between LE and CT excitations according to the original paper on Δr^6^. The D index values also support this finding, as shown in Table 2, where the distances between the centers of the hole and electron isosurfaces, i.e., Chole and Cele centroids, are considered very close to each other for all excitations, with small D index values for all excited states. These results provide evidence for LE excitations.

The Sr index is examined, and it is found that the Sr indices for all excited states are relatively large due to the low value of the D index. Also, in the S0 → S10, S0 → S15, and S0 → S20 transition, the Sr value is high, reaching 0.99. This is because an overlapping between the hole and electron is observed in the S10, S15, and S20 excited states compared to the other studied excited states S1, and S5 (See Table 2). The H index is a measure of the width of the average distribution of the hole and electron in an excited state. Analysis of the values in Table 2 reveals that the density distributions of both the holes and electrons for all excited states are similar, with the H index values being relatively close to each other. This suggests that the degree of charge transfer and delocalization in the excited states is comparable, regardless of the specific system under consideration. The t indices for the excitations from S0 to the other excited states are negative, which suggests a very low degree of separation between their holes and electrons in S1, S5, S10, S15, and S20, making it more reasonable to consider them as LE excitations. In contrast, the hole and electron distributions of the S0 → S1/S5/S10/S15/S20 transitions are more delocalized, as reflected by their relatively small HDI and EDI values.

### Charge transfer

**Figure 8:**
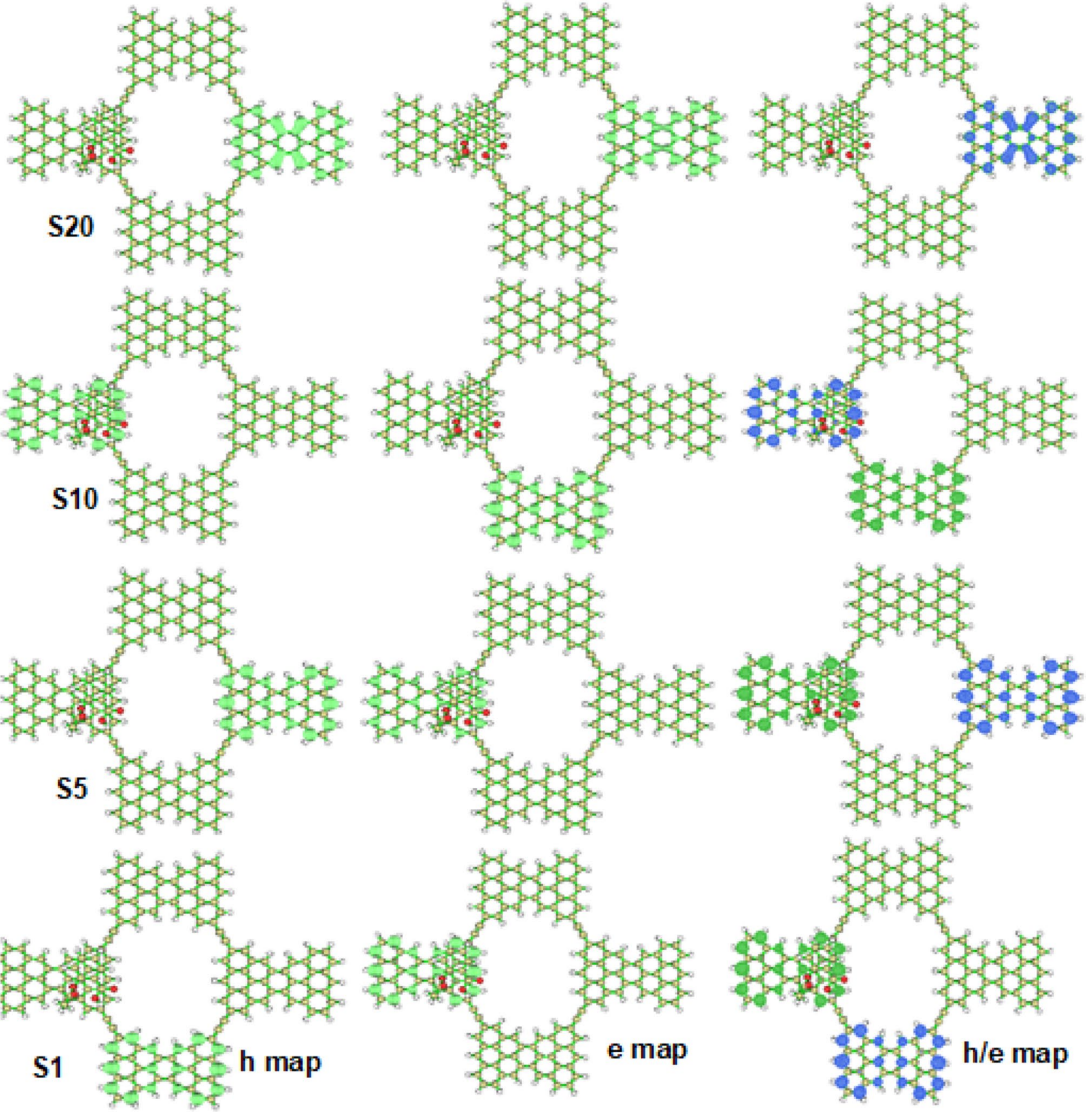
Hole (h), electron (e), and hole/electron distribution maps forCGM1-aspirin in S1, S5, S10, and S20 excited states.

Table 3 displays the percentage charge distribution of holes, electrons, and pharmaceutical pollutants' charge transfer in the form of CGM1/aspirin, CGM1/ibuprofen, CGM1/diclofenac, and CGM1/paracetamol. These results are obtained through Multiwfn software and presented in Table 3. Furthermore, maps illustrating the distribution of holes, electrons, and their mixing in CGM1-aspirin, CGM1-ibuprofen, CGM1-diclofenac, and CGM1-paracetamol in S1, S5, S10, and S20 excited states are generated using Multiwfn software and presented in Figs. 8, S1, S2, and S3. The hole and electron maps are displayed independently because, in certain excited states, there may be a significant overlap between the distributions of the holes and electrons. Therefore, to provide clarity and avoid confusion, the two maps are presented separately. The CT(%) values in Table 3 represent the percent of charge transfer character between CGM1 and aspirin, ibuprofen, diclofenac, and paracetamol. These values can be determined by subtracting the percentage of CGM1 in the hole (hole(CGM1%)) from that in the electron (ele(CGM1%)). The obtained results are presented in Table 3. Based on the results in Table 3, several conclusions can be drawn. First, for CGM1-aspirin, the percentage of holes and electrons in the S1 excited state is 100%, indicating no charge transfer between CGM1 and aspirin. Similarly, in S10 excited state, 100% of the charge transfer is in CGM1. This is confirmed by the value of CGM1/aspirin CT(%) = 0%. Second, high percentages of hole and electron distributions and low values of CGM1/aspirin CT(%) are observed in S5, S10, and S15 for CGM1-aspirin, S5, S10, S15, and S20 for CGM1-ibuprofen, S15 and S20 for CGM1-diclofenac, and S5, S10, and S15 for CGM1-paracetamol. This indicates that a high percentage of charge transfer occurs in CGM1, while a small percentage occurs between CGM1 and the pharmaceutical pollutants. Third, high values of either %hole and low %electron or vice versa result in high values of CGM1-drug CT%. This is observed in S1 for CGM1-ibuprofen and CGM1-paracetamol and in S1, S5, and S10 for CGM1-diclofenac. This leads to a small percentage of CGM1/CGM1 CT and a high percentage of CT between CGM1 and ibuprofen, diclofenac, and paracetamol. Finally, the positions of hole and electron distributions vary when moving from one excited state to another or from one chemical structure to another. This dependence on the type of excited state order and the chemical composition of the complex is confirmed by the hole, electron, and ratio maps presented in Figs. 8, S1, S2, and S3.

**Table 3:** Charge transfer investigations between CGM1 and pharmaceutical pollutant drugs.

Complexes	Electronic transitions	Hole (CGM1%)	Ele (CGM1%)	CGM1/aspirin CT (%)
CGM1/aspirin	S0 → S1	100%	100%	0%
	S0 → S5	100.00%	99.79%	0.21%
	S0 → S10	99.72%	100%	− 0.28%
	S0 → S15	99.87%	99.88%	− 0.01%
	S0 → S20	100%	100%	0.00%
		Hole (ibuprofen%)	Ele (ibuprofen%)	CGM1/ibuprofen CT (%)
CGM1/ibuprofen	S0 → S1	100.11%	34.43%	65.68%
	S0 → S5	100.05%	99.94%	0.11%
	S0 → S10	100.01%	99.97%	0.04%
	S0 → S15	88.80%	89.35%	− 0.55%
	S0 → S20	99.98%	100.01%	− 0.03%
		Hole (diclofenac%)	Ele (diclofenac%)	CGM1/diclofenac CT (%)
CGM1/diclofenac	S0 → S1	100.00%	34.76%	65.24%
	S0 → S5	37.22%	100.10%	− 62.88%
	S0 → S10	31.64%	100.02%	− 68.38%
	S0 → S15	76.04%	77.16%	− 1.12%
	S0 → S20	99.74%	99.82%	− 0.08%
		Hole (paracetamol%)	Ele (paracetamol%)	CGM1/paracetamol/CT (%)
CGM1/paracetamol	S0 → S1	33.01%	100.05%	− 67.04%
	S0 → S5	99.92%	100.00%	− 0.08%
	S0 → S10	100.03%	99.96%	0.07%
	S0 → S15	72.79%	70.11%	2.68%
	S0 → S20	45.98%	39.70%	6.28%

In conclusion, the design of the new porous Clar's Goblet (CGM1) structure shows great promise as a highly sensitive sensor for pharmaceutical pollutants, specifically the painkiller drugs aspirin, paracetamol, ibuprofen, and diclofenac. The research conducted on the electronic, adsorption, and optical properties of CGM1 with these drugs provides valuable insights into the potential applications of this material. The observed red shift in the absorption spectra of CGM1-drug complexes confirms the successful adsorption of the painkillers onto the surface of CGM1. Computational chemistry analysis of excited states in the CGM molecule system using various indices provides valuable insights into the electronic structure and properties of molecules. The results indicate predominantly local excitations based on low Δr indices and small D index values. The Sr indices suggest overlapping between hole and electron distributions in certain excited states. The H index values show comparable degrees of charge transfer and delocalization. Non-covalent interaction analysis reveals the presence of van der Waals interactions in the complexes formed between pollutant drugs and the CGM1 substrate. These findings enhance our understanding of excited state properties and interactions, contributing to the broader field of computational chemistry. These findings highlight the significance of CGM1 as a promising candidate for the detection and removal of pharmaceutical pollutants from the environment.

### Ethical approval

This article does not contain any studies involving animals performed by any of the authors.

### Consent to participate

This article does not contain any studies involving animals performed by any of the authors.

### Supplementary Information

Supplementary Figures.

## Data Availability

All data generated or analyzed during this study are included in this published article.
