# Supplementary material for: Promising sensors for pharmaceutical pollutant adsorption using Clar’s goblet-based 2D membranes
Source: Sci Rep. 2024 Jan 9;14:889. doi: 10.1038/s41598-023-50802-0 (PMC10776697; doi:10.1038/s41598-023-50802-0)
Supplement: Supplementary file 1 — Supplementary Figures. [file 41598_2023_50802_MOESM1_ESM.docx]

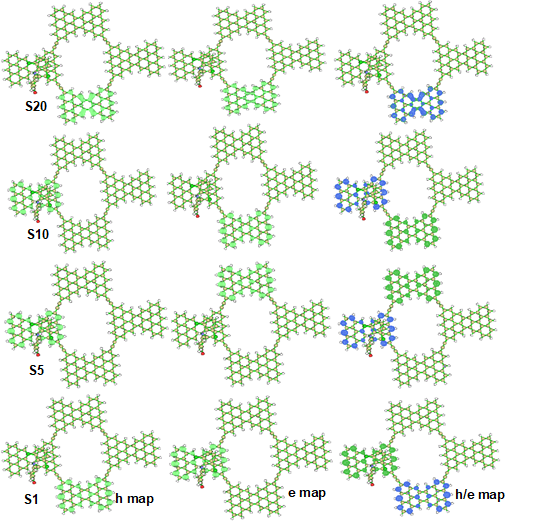


Fig. S1. Hole(h), electron (e), and hole/electron distribution maps of CGM1-diclofenac in S1, S5, S10, and S20 excited states


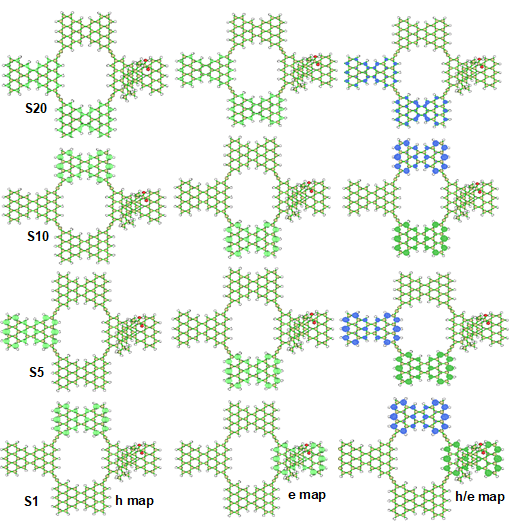


Fig. S2. Hole(h), electron (e), and hole/electron distribution maps of CGM1-ibuprofen in S1, S5, S10, and S20 excited states


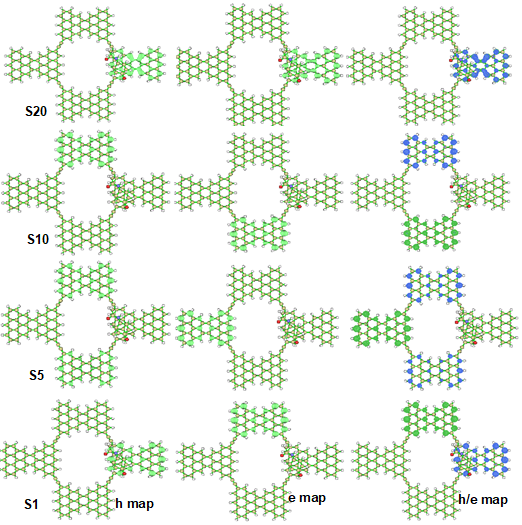


Fig. S3. Hole(h), electron (e), and hole/electron distribution maps of CGM1-paracetamol in S1, S5, S10, and S20 excited states
